# Risk factors of daptomycin overexposure: a case-control study

**DOI:** 10.1128/aac.01139-25

**Published:** 2025-11-07

**Authors:** Clotilde Vellat, Romain Garreau, Aurélien Millet, Catherine Piron, Laurent Bourguignon, Sandrine Roux, Tristan Ferry, Sylvain Goutelle, on behalf of the Lyon Bone and Joint Infection Study Group

**Affiliations:** 1 Laboratoire de Biométrie et Biologie Evolutive, UMR 5558, CNRS & Université Lyon 1, Villeurbanne, France; 2 Hospices Civils de Lyon, Groupement Hospitalier Nord, Service de Pharmacie 148706 , Lyon, France; 3 Univ Lyon, Université Claude Bernard Lyon 1, Facultés de Médecine et de Pharmacie de Lyon, Lyon, France; 4 Hospices Civils de Lyon, Groupement Hospitalier Sud, Service de Biochimie et Biologie Moléculaire, UM Pharmacologie-Toxicologie, Lyon, France; 5 Hospices Civils de Lyon, Groupement Hospitalier Nord, Hôpital de la Croix-Rousse, Service des Maladies Infectieuses et Tropicales, Centre de Référence pour la Prise en Charge des Infections Ostéo-Articulaires Complexes (CRIOAc Lyon), Lyon, France; Providence Portland Medical Center, Portland, Oregon, USA

**Keywords:** bone joint infection, therapeutic drug monitoring, pharmacokinetics, daptomycin

## Abstract

In our institution, therapeutic drug monitoring of daptomycin is performed routinely and cases of high trough concentrations have been observed in patients without known risk factors. The aim of this study was to identify risk factors of daptomycin overexposure. We performed a case-control study of daptomycin overexposure in patients who received daptomycin between 2013 and 2021. Cases and controls were defined as patients with trough concentration (Cmin) ≥60 mg/L and Cmin <60 mg/L, respectively. Univariate and multivariate analyses were performed with logistic regression models. Retained variables were further analyzed by subgroup analysis and comparison of the pharmacokinetic parameters of daptomycin. We analyzed data from 78 and 26 patients in the control and case groups, respectively. The male-to-female ratio was 1.5. The median (interquartile range) of age, body weight, and creatinine clearance was 66.5 (55–77) years, 77 (65–96) kg, and 98.5 (53–124) mL/min, respectively. Increasing body mass index (BMI) and co-administration of irbesartan were identified as risk factors of daptomycin overexposure with odds ratio (OR) (95% confidence interval [CI]) of 2.9 [1.4–6.2], and 6.1 [1.1–40.8], respectively, whereas increasing creatinine clearance was associated with decreasing risk, with OR of 0.16 [0.05–0.35]. The influence of BMI was attributed to the non-linear relationship between body weight and daptomycin PK parameters and the use of weight-based dosing in patients with high BMI. In addition to renal impairment, high BMI and irbesartan co-administration may be associated with an augmented risk of daptomycin overexposure. Dosing based on actual body weight should be avoided in obese patients.

## INTRODUCTION

Daptomycin has a rapid bactericidal effect against a broad spectrum of Gram-positive bacteria (1). Its effectiveness against resistant bacteria, such as Methicillin-resistant *Staphylococcus aureus* and Vancomycin-resistant *Enterococcus,* as well as its good safety profile (less nephrotoxicity than vancomycin), makes it an attractive option to treat severe Gram-positive infections.

Daptomycin exposure is predictive of its antibacterial effect. The ratio of area under the concentration-time curve over the MIC (AUC/MIC) is considered the most relevant pharmacokinetic (PK)/pharmacodynamic (PD) index of daptomycin efficacy (2, 3). While the most commonly reported adverse effects are generally mild and reversible upon discontinuation of treatment, daptomycin may be responsible for severe muscle toxicity and eosinophilic pneumonia (4–6). It has been shown that high concentration of daptomycin is a risk factor for those two adverse events (5).

Significant inter-individual and intra-individual PK variability has been observed (7). Renal function and body weight influence daptomycin dosage requirements, and dosing based on actual body weight is recommended in the drug label. While the approved daptomycin dosage is 4–6 mg/kg, real-life data indicate that higher dosages (8 to 12 mg/kg) are widely used currently, especially for treating severe or deep-seated infections, such as bone and joint infection (BJI), an off-label indication (8, 9). Both approved and high-daptomycin dosages may be associated with low rates of optimal exposure because of PK variability (10–12). Therapeutic drug monitoring (TDM) of daptomycin has been suggested to individualize dosage. This approach aims at minimizing toxicity risks, optimizing treatment efficacy, and reducing the risk of resistance emergence associated with subtherapeutic doses (5, 13–15).

In our institution, daptomycin TDM is available in routine and used to individualize daptomycin dosage, especially in patients with BJI. Over the past years, we observed several cases of high daptomycin trough concentrations in patients with no known risk factors of overexposure, such as renal impairment. Other risk factors might exist, but those have not been clearly identified so far.

This retrospective, case-control study aimed to identify the risk factors associated with daptomycin overexposure.

## MATERIALS AND METHODS

### Data collection

We performed a retrospective, observational case-control study. The case group was identified from all patients hospitalized in the infectious disease department between 01 January 2013 and 31 December 2021, who were administered daptomycin. The other inclusion criteria were as follows: age ≥18 years, no renal replacement therapy, and measurement of a Cmin above the overexposure threshold set at 60 mg/L (see below) on the first TDM occasion. The sampling time defining an acceptable minimal concentration was set at less than 3 h before re-administration. Patients in the control group were identified from a cohort of 1,130 patients followed at the reference center for complex bone and joint infections in Lyon (CRIOAc: https://www.crioac-lyon.fr/en) who were administered daptomycin over the same period. Controls with the same inclusion criteria (except Cmin ≥ 60 mg/L) were randomly selected in this cohort with a 3:1 ratio.

Therapeutic drug monitoring of daptomycin was performed at least once for each patient. Each daptomycin TDM occasion typically included measured plasma concentrations at three time points: before the next infusion (Cmin, h = 0), 30 min after the end of the infusion (Cmax), and a last sample obtained 5 to 6 h post-infusion. Daptomycin concentration was determined by high-performance liquid chromatography with ultraviolet detection assay with a photodiode array detector until 2020 and then high-performance liquid chromatography-tandem mass spectrometry (methods cross-validated), as described elsewhere (7, 10). The lower limit of quantification was 2 mg/L. The interday imprecision was less than 11% with a bias lower than 8%.

Variables collected included anthropometric data, such as sex, age, height, weight, and body mass index (BMI). Biological data were also collected, including serum creatine phosphokinase (CPK), serum C-reactive protein (CRP), serum creatinine, and serum protein. Creatinine clearance (CL_Creat_) was estimated using the Cockcroft-Gault formula. Therapeutic data, including daptomycin dosing history, measured Cmin, and all co-treatments, were also collected. Co-prescribed drugs were classified according to the pharmacological and chemical subgroups (third level) of the WHO Anatomical Therapeutic Chemical (ATC) classes (16).

### Definition of overexposure

A daptomycin Cmin >24.3 mg/L has been associated with an increased risk of CPK elevation in the study by Bhavnani et al. that was performed in patients who received 6 mg/kg daily (5). However, it has been shown that many patients who were administered a daptomycin dose of 8–12 mg/kg/24 h had Cmin higher than this threshold, and most of them have no sign of toxicity (5, 17–19). This means that although achieving Cmin <24.3 mg/L is desirable for safety, this cut-off is not a relevant value to define a high, unexpected exposure with current dosing practice.

The distribution of daptomycin Cmin at steady state was obtained by simulating a daily daptomycin administration of 10 mg/kg for 14 days in a typical subject weighing 70 kg and having a creatinine clearance of 90 mL/min. The simulation was performed using BestDose software (20), based on a previously published population PK model of daptomycin (21). This distribution is shown in Fig. S1. The probability of a Cmin >60 mg/L (which corresponds to an average AUC of 2,470 mg·h/L over 24 h based on the PK model) was 5.7%, which corresponds approximately to the 95th percentile of the Cmin distribution and so Cmin > 60 mg/L was defined as the overexposure cut-off in the subsequent analysis.

### Statistical analysis

#### Exploratory data analysis

Missing data were identified and imputed using a multiple imputation by chained equation (mice) with a random forest algorithm for data missing at random. Post-imputation checks were conducted to confirm that no unintended imputations occurred and that the resulting distributions were realistic, without any outlier values.

Data distributions were checked for normality using the Shapiro-Wilk test. In the case of deviation from normal distribution, comparisons were performed using the Wilcoxon signed-rank test for continuous data and Fisher’s exact test for categorical data between the case and control groups. All tests were two-sided. The BMI was analyzed as both a continuous and categorical variable, based on stages defined by the WHO: underweight for BMI <18.5 kg/m²; normal for BMI within 18.5–24.9 kg/m²; overweight for BMI within 24.9–29.9 kg/m²; obese for BMI ≥30 kg/m² (22). Scatter plots were done to visualize the relationship between daptomycin Cmin and other variables.

#### Logistic regression analysis

The probability of daptomycin overexposure was further analyzed by univariate and multivariate logistic regression. Co-administered drugs were examined as categorical data. Log transformation was applied to continuous variables in the case of a non-Gaussian distribution. Then, all continuous variables were normalized by the mean cohort value prior to regression analysis. Variance inflation factor (VIF) was applied to identify collinearity among variables.

The effect of each explanatory variable on the target variable was tested using the Wald test. Variables associated with a *P* value <0.2 in the univariate regression analysis were selected for the multivariate analysis. Model selection was performed by using both forward addition and backward deletion. The best model was selected based on the Akaike Information Criterion (AIC), and statistical significance was defined as a Δ_AIC_ ≥1.8 between the two models (23–25).

The model performance was also evaluated using receiver operating characteristic (ROC) curve analysis with calculation of the area under the ROC curve. The 95% confidence interval for the area under the ROC curve was obtained through a bootstrap procedure with the final model (*n* = 1,000 replicates). Furthermore, due to the group imbalance, a precision-recall curve was also used to assess the model’s performance. The influence of the variables was assessed using a variable importance plot (VIP), where the importance of each variable was computed using the permutation importance method.

#### Analysis of daptomycin dosages and PK parameters

Retained variables in the regression models were further analyzed with subgroup analyses. First, patients were classified based on their BMI. The number of cases with normal renal function, as well as the means (SD) of daptomycin Cmin, dose per total body weight (TBW), dose per ideal body weight (IBW), and dose per adjusted body weight (AjBW) were calculated and compared between the different BMI categories using the Kruskal-Wallis rank-sum test.

The subjects' individual PK parameters were estimated from TDM data by using a previously published population PK model of daptomycin (26). This model was developed in 183 patients (106 men, 77 women) with bone and joint infection. PK parameter values are shown in Table S2. Wilcoxon’s and Kruskal-Wallis tests were used to compare the distributions of each PK parameter between case and control groups; BMI groups; and groups defined by co-administered drugs.

All statistical analyses were performed using R software (Version 4.4.1 R Core Team [2024]).

Pharmacokinetic modeling was performed using Monolix (Version 2023R1; Lixoft, Antony, France).

## RESULTS

### Characteristics of case and control groups

Twenty-eight patients had daptomycin Cmin >60 mg/L over the study period. After reviewing their medical records, 26 subjects were included in the case group. Two subjects were excluded because sampling for daptomycin concentration measurement was performed too early (<21 h after the last administration). After excluding patients who did not meet the selection criteria, 78 patients out of the 1,130 patients treated with daptomycin at the CRIOAC were randomly selected in the control group.

**Fig 1:**
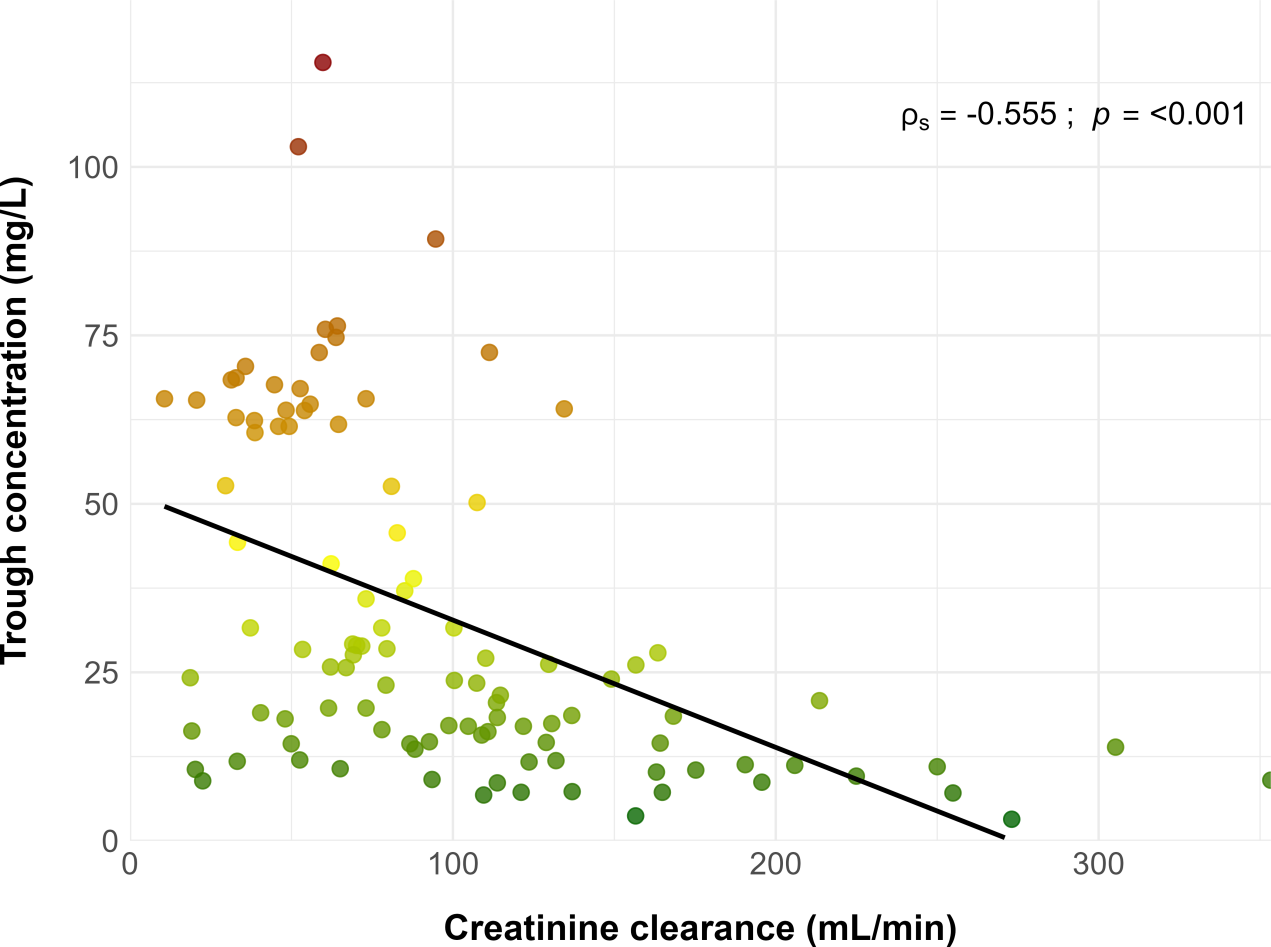
Correlation between daptomycin trough concentration and creatinine clearance. The black line represents the regression line. The color scale of dots reflects the level of trough concentrations from the lowest (green) to the highest (dark red). The ρs represents Spearman’s correlation coefficient, and *P* represents the associated *P* value.

**Fig 2:**
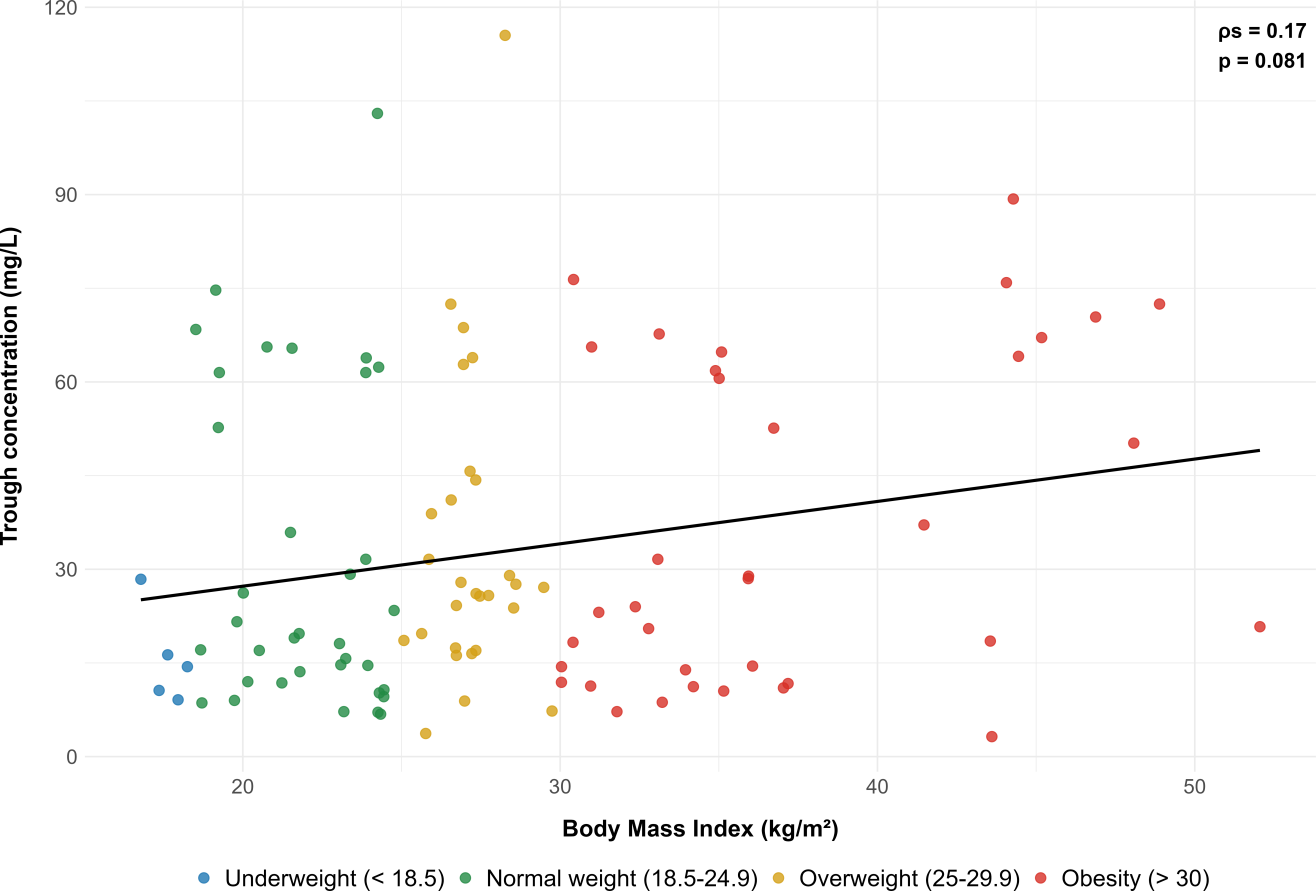
Correlation between daptomycin trough concentration and body mass index. The black line represents the linear regression line. The ρ_s_ represents Spearman’s correlation coefficient, and *P* represents the associated *P* value.

Patients’ characteristics in the case and control groups are presented in Table 1. Compared with the control group, patients in the case group were significantly older, with a median age (interquartile range) of 75 (68, 81) versus 62 (52, 73) years. Their serum creatinine level was higher, and their creatinine clearance was about twofold lower, 68 (51, 93) versus 124 (92, 151) mL/min. They also displayed lower height and higher CRP levels. Other characteristics were similar between the two groups, including daptomycin median dosage, which was approximately 8 mg/kg in both groups. Scatter plots showed a significant negative correlation between Cmin and creatinine clearance and a limited positive correlation between Cmin and BMI, as shown in Fig. 1 and 2, respectively.

**TABLE 1:** Patients’ characteristics in case and control groups^*e*^^,^^*f*^

Characteristics	Case patients	Control patients	*P* value^*a*^	Corrected *P* value^*b*^
	*n* = 26	*n* = 78		
Sex			>0.9	>0.9
Female	11 (42%)	32 (41%)		
Male	15 (58%)	46 (59%)		
Age (years)	75 (68, 81)	62 (52, 73)	<0.001	0.009
Height (cm)	165 (158, 172)	172 (162, 176)	0.039	0.2
Weight (kg)	76 (68, 99)	78 (64, 96)	0.6	>0.9
BMI (kg/m²)	28 (24, 35)	27 (23, 31)	0.2	0.6
CRP (mg/L)	26 (14, 54)	11 (5, 29)	0.005	0.04
Serum protein (g/L)	70 (65, 74)	72 (68, 76)	0.086	0.3
Serum creatinine (µmol/L)	124 (92, 151)	68 (51, 93)	<0.001	<0.001
Creatinine clearance (mL/min)^*c*^	52 (39, 63)	103 (69, 137)	<0.001	<0.001
Daptomycin dose (mg/kg total body weight)	8.33 (7.74, 9.76)	8.24 (7.28, 9.31)	0.3	0.8
TDM occasion/subject (mean)	1.08	1.83		
Measured daptomycin concentration (n)	55	345		
Peak (n)^*d*^	14	116		
Concentration (mg/L)	152.5 (131, 173)	78 (65, 91)		
Trough (n)^*d*^	28	125		
Concentration (mg/L)	68 (64, 76)	16.5 (12, 26)		
Others (n)	13	104		
Concentration (mg/L)	121 (111, 127)	50 (41, 64)		
AUC over 24 h (mg.h/L)	2,460 (2,333, 2,931)	993 (805, 1,296)		

^*a*^ Wilcoxon test, Pearson’s Chi-square test.

^*b*^ Corrected by Benjamini-Hochberg method.

^*c*^ Creatinine clearance was estimated using the Cockcroft-Gault formula.

^*d*^ Peak concentrations were sampled 30 min after the end of the infusion, and trough concentrations were sampled less than 3 h before re-administration.

^*e*^ Data are expressed as median (IQR) or number (%).

^*f*^ BMI, body mass index; CRP, C-reactive protein; TDM, therapeutic drug monitoring.

The co-treatments classified by ATC groups in case and control patients are shown in Table S1. The proportions of irbesartan, beta-blockers, antifungals, other antihypertensive agents, laxatives, hypokalemic diuretics, calcium channel blockers, tetracyclines, proton pump inhibitors (PPIs), other lipid-lowering agents, antiplatelet agents, hyperkalemia treatments, and thyroid medications were different between the two groups. Laxatives, antifungals, other antihypertensives, other lipid-lowering agents, and thyroid medications were excluded from further analyses as they were not considered clinically relevant risk factors of daptomycin overexposure.

### Risk factors of overexposure

Among the 104 subjects in the data set, eight had at least one missing variable value. A total of 10 values were missing: five for CRP, two for serum protein, one for weight, one for BMI, and one for CL_Creat_. Data distribution after imputation was adequate (data not shown).

Results from the logistic regression analysis are shown in Table 2. In the univariate analysis, the probability of daptomycin overexposure significantly increased with increasing age, CRP, and BMI and with decreasing creatinine clearance and serum protein level. The analysis of drug therapy data showed that PPIs, beta-blockers, hypokalemic diuretics, and irbesartan administration were significantly associated with daptomycin overexposure (*P* < 0.1).

**TABLE 2:** Logistic regression analysis of the probability of daptomycin overexposure^*c*^

Variable	OR^*a*^	95% CI	*P* value^*b*^
Univariate analysis			
Daptomycin dose (mg/kg)	1.33	0.86–2.09	0.2
CL_Creat_ (mL/min)	0.31	0.17–0.54	<0.001
Age (years)	2.84	1.58–5.68	<0.001
BMI (kg/m²)	1.5	0.97–2.33	0.07
CRP (mg/L)	1.77	1.13–2.80	0.01
Serum protein (g/L)	0.62	0.37–0.97	0.04
Irbesartan use	5.95	1.35–30.99	0.02
Beta-blockers use	4.97	1.97–13.17	<0.001
PPI use	2.3	0.94–5.81	0.07
Diuretics use	2.44	0.95–6.28	0.06
Other lipid-lowering drugs used	6.42	0.59–141	0.14
Multivariate analysis			
Intercept	0.082	0.02–0.22	<0.001
CL_Creat_ (mL/min)	0.156	0.05–0.36	<0.001
BMI (kg/m²)	2.893	1.48–6.16	<0.001
Beta-blockers	3.042	0.94–10.45	0.066
Irbesartan	6.108	1.04–40.87	0.047
Dose/kg	1.568	0.94–2.74	0.09

^*a*^ OR represents the effect of a one-standard-deviation (SD) change in the predictor variable. One SD corresponds to 64.31 mL/min for CLcreat and 7.9 kg/m² for BMI.

^*b*^ Wald test.

^*c*^ BMI, body mass index; CI, confidence interval; CRP, C-reactive protein; CL_Creat_, creatinine clearance; OR, odds ratio; PPI, proton pump inhibitors.

In the multivariate analysis, only creatinine clearance, BMI, and irbesartan remained significant predictors of overexposure. This result was confirmed on the VIP shown in Fig. S2.

**Fig 3:**
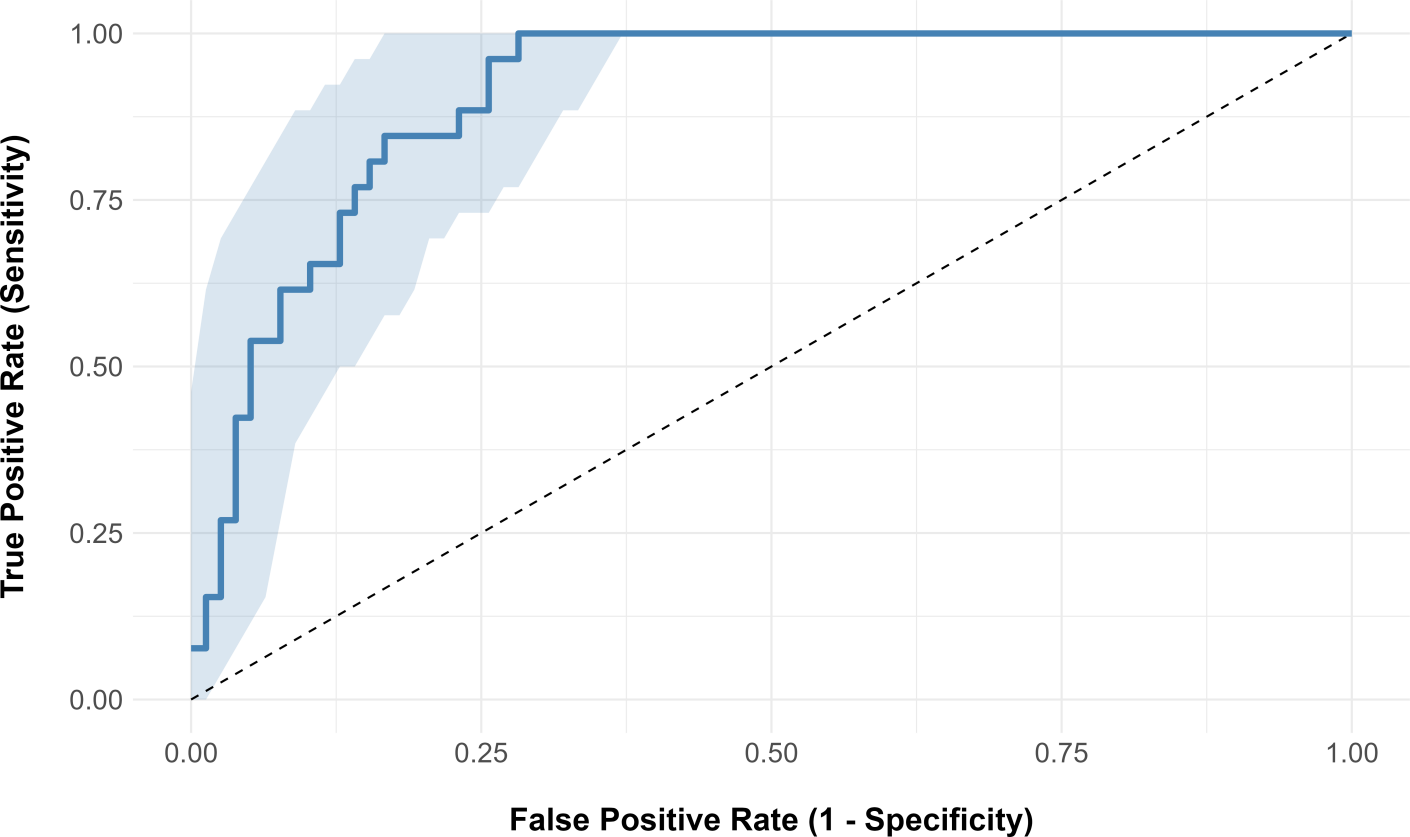
Receiver operating characteristic curve for classification of overexposure by the logistic regression model. The blue line represents the ROC curve, the blue area represents the 95% confidence interval, and the dashed line represents the random classification.

The predictive performance of the multivariate logistic regression model is illustrated in the plot of the ROC curve (Figure 1 and 3) and the precision-recall curve (Fig. S3). The area under the ROC curve was 0.91 (95% CI, 0.84–0.95), while the precision-recall curve AUC was 0.707. All variables had VIF values below 2, suggesting that significant multicollinearity was not present in the final model (data not shown).

### Daptomycin dosing and PK in relation to BMI

**Fig 4:**
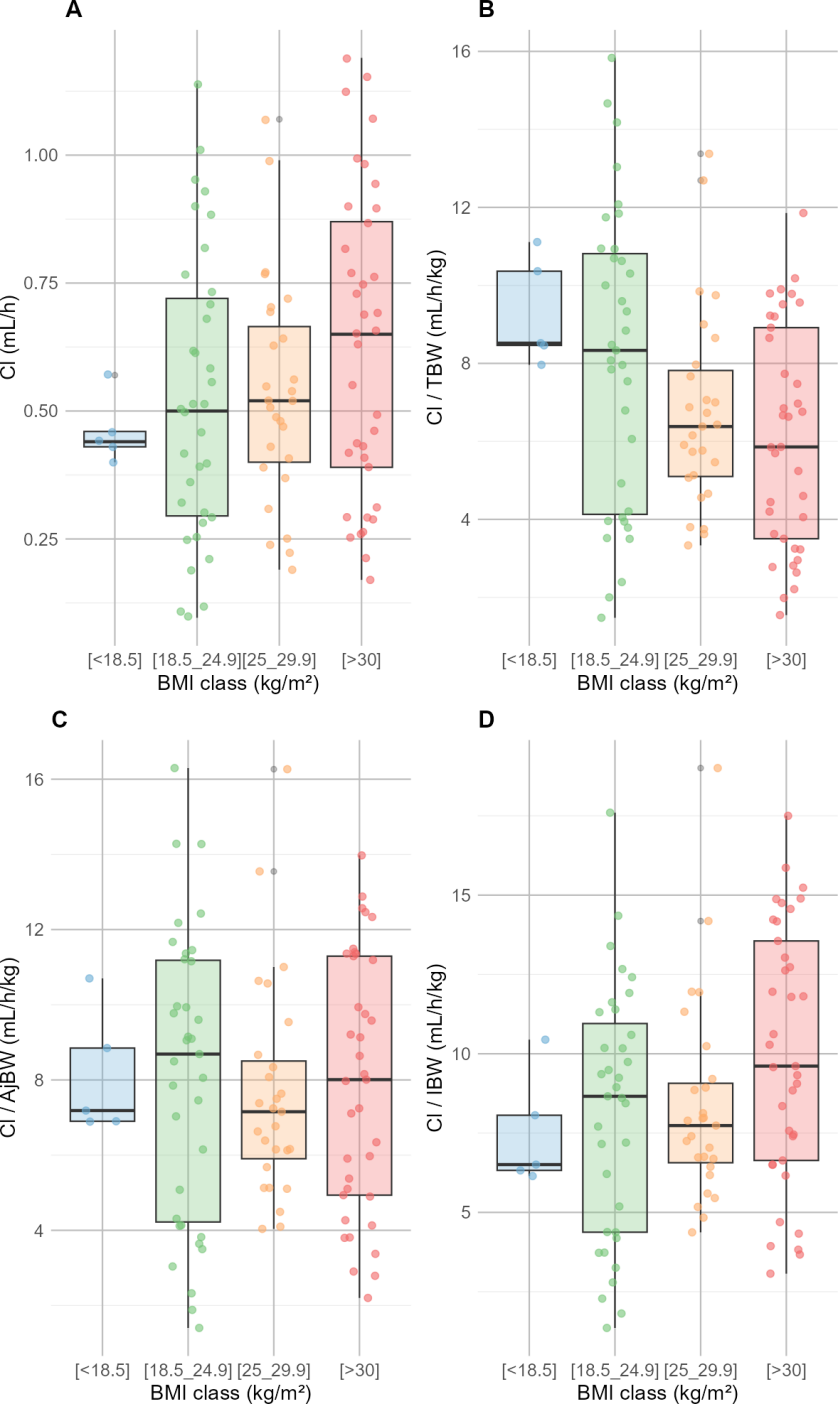
Distribution of daptomycin clearance in groups of body mass index. Panels show total daptomycin clearance (**A**), daptomycin clearance/total body weight (**B**), daptomycin clearance/adjusted body weight (**C**), and daptomycin clearance/ideal body weight (**D**).

The rationale of BMI as a risk factor of daptomycin overexposure was further examined. Daptomycin doses expressed per kilogram of various weight descriptors, along with daptomycin PK parameters across BMI groups, are displayed in Table 3. Daptomycin doses indexed on total body weight were not different between groups. By contrast, doses indexed on AjBW and IBW were significantly different between BMI groups, and patients with the highest BMI showed the highest doses. In addition, daptomycin total body clearance (CL_Dap_) increased with BMI, while CL_Dap_ indexed on total body weight decreased, and CL_Dap_ indexed on ideal body weight or adjusted body weight was comparable across BMI categories, as shown in Fig. 4.

**TABLE 3:** Daptomycin dosage, case, and PK in BMI subgroups^*d*^

BMI class (kg/m²)	[<18.5]	[18.5–24.9]	[25–29.9]	[≥30]	*P* value^*b*^
	*n* = 5^*a*^	*n* = 35^*a*^	*n* = 27^*a*^	*n* = 37^*a*^	
Daptomycin concentration (mg/L)	16 (8)	31 (25)	34 (24)	37 (26)	0.2
Dose/TBW (mg/kg)	8.47 (1.49)	8.68 (1.60)	8.58 (1.83)	7.98 (1.45)	0.2
Dose/IBW (mg/kg)	6.8 (8)	8.7 (1.7)	10.6 (3.2)	13.7 (3.7)	<0.001
Dose/AjBW (mg/kg)	7.37 (1.49)	8.66 (1.62)	9.69 (2.38)	10.57 (2.13)	<0.001
Case (n)	0	9 (26%)	5 (18%)	12 (33%)	0.4
PRF cases^*c*^ (n)	0	1 (4.8%)	0	8 (25%)	0.032
Cl_Dap_ (L/h)	0.46 (0.07)	0.52 (0.28)	0.53 (0.22)	0.63 (0.30)	0.4
Cl_Dap_/TBW (mL/h/kg)	9.3 (1.4)	8.1 (3.9)	6.8 (2.5)	6.1 (2.9)	0.026
Cl_Dap_/IBW (mL/h/kg)	7.5 (1.8)	8.2 (4.0)	8.3 (3.2)	10.0 (4.0)	0.13
Cl_Dap_/AjBW (mL/h/kg)	8.1 (1.7)	8.1 (3.9)	7.6 (2.9)	7.9 (3.4)	0.9

^*a*^ All values are given as Mean (SD).

^*b*^ Kruskal-Wallis rank sum test and Fisher’s exact test.

^*c*^ Cases in patients with preserved renal function, i.e., normal or mildly impaired renal function population (with creatinine clearance ≥60 mL/min).

^*d*^ AjBW, adjusted body weight; BMI, body mass index; Cl_Dap_, daptomycin clearance; PRF, correct renal function; IBW, ideal body weight; TBW, total body weight.

The proportions of overexposure across BMI groups were not significantly different. Yet, the highest proportion of overexposed patients was in the group with BMI >30 kg/m², accounting for 46% of the cases, even though this BMI category represented only 35% of the subjects. Of note, in patients with overexposure and CL_Creat_ >60 mL/min, 8 out of 9 were obese.

Pharmacokinetic parameters in patients’ subgroups defined by BMI and irbesartan co-administration are displayed in Table 4. Daptomycin median clearance was 2.4-fold lower in cases compared to controls, and twofold lower in patients who were co-administered irbesartan (*P* = 0.031). This difference in daptomycin PK was not observed for other angiotensin II receptor antagonists, as shown in Fig. S4.

**TABLE 4:** Daptomycin pharmacokinetics parameters in subgroups defined by BMI and co-administration of irbesartan^*d*^

	CL [L/h]	V1 [L]	Q [L/h]	V2 [L]
Case vs controls				
Case, *n* = 26^*a*^	0.26 (0.21, 0.30)	8.17 (6.44, 9.15)	0.55 (0.52, 0.57)	3.71 (3.37, 4.06)
Control, *n* = 78^*a*^	0.63 (0.47, 0.81)	8.11 (6.65, 10.47)	0.55 (0.49, 0.61)	3.50 (2.92, 3.92)
*P* value^*b*^	0.001	0.5	0.7	0.056
Body mass index groups				
[<18.5], *n* = 5^*a*^	0.44 (0.43, 0.46)	6.73 (6.67, 8.46)	0.49 (0.47, 0.51)	3.02 (2.63, 4.01)
[18.5–24.9], *n* = 35^*a*^	0.50 (0.30, 0.72)	6.73 (6.06, 8.82)	0.56 (0.50, 0.60)	3.43 (3.15, 3.90)
[24.9–29.9], *n* = 27^*a*^	0.52 (0.40, 0.67)	8.68 (7.38, 9.41)	0.57 (0.54, 0.61)	3.54 (3.29, 3.89)
[>30], *n* = 37^*a*^	0.65 (0.39, 0.87)	8.58 (7.45, 10.71)	0.55 (0.48, 0.59)	3.67 (3.04, 3.92)
*P* value^*c*^	0.4	0.028	0.4	0.8
Irbesartan co-administration				
Yes, *n* = 8^*a*^	0.27 (0.19, 0.48)	6.40 (5.71, 7.78)	0.54 (0.43, 0.59)	3.37 (3.00, 3.81)
No, *n* = 96^*a*^	0.52 (0.39, 0.75)	8.30 (6.67, 9.74)	0.55 (0.51, 0.60)	3.59 (3.10, 3.93)
*P* value^*b*^	0.031	0.084	0.4	0.8

^*a*^ All data are expressed as median (IQR).

^*b*^ Wilcoxon rank sum test.

^*c*^ Kruskal-Wallis rank sum test.

^*d*^ CL_Creat_, creatinine clearance; V1, central compartment volume; Q, intercompartmental clearance; V2, peripheral compartment volume.

## DISCUSSION

It has been shown that some adverse reactions to daptomycin, myotoxicity, and eosinophilic pneumonia are concentration-dependent in part. Thus, it is relevant to identify risk factors of overexposure. To our knowledge, this is the first study to investigate drug-related determinants of daptomycin overexposure. In our study, the primary risk factors of daptomycin overexposure, defined as Cmin >60 mg/L, were impaired renal function, obesity, and the concomitant use of irbesartan.

Daptomycin is primarily eliminated unchanged in the urine. Impaired renal function results in reduced drug clearance and daptomycin accumulation (7, 27–29). As expected, renal function had the greatest impact on the risk of overexposure in our results, aligning with previously published pharmacokinetic data and the drug label. However, our data show that overexposure can occur even in patients with normal or mildly impaired renal function, which suggests the role of other determinants.

The relationship between higher BMI and increased risk of overexposure was more surprising. While the linear correlation between Cmin and BMI was not significant (Fig. 2), the multivariate logistic regression analysis showed that BMI was predictive of overexposure. Renal clearance and drug distribution are frequently altered in the obese population (30). It has been demonstrated that obese patients exhibit distinct PK profiles compared to those with normal weight, and daptomycin PK could be affected by these alterations (31, 32). Dvorchik et al. have reported increased values of daptomycin CL and Vd in obese patients compared with patients with normal weight (33). Indeed, the positive correlation between daptomycin PK parameters and weight is the main rationale for weight-based dosing. Thus, one could expect similar or lower, not higher, concentration of daptomycin in obese patients. This can be explained by the non-linear relationship between daptomycin clearance and total body weight in obese patients, as well as dosing based on total body weight. Dvorchik et al. demonstrated that daptomycin CL and Vd increased with total body weight, but less than proportionally, and these parameters were lower in obese patients when expressed per kg of body weight (33). Our findings are consistent with these observations. Therefore, daptomycin dosing based on total body weight results in greater exposure in obese patients. Pai et al. reported that Cmax and AUC values were 60% higher in morbidly obese subjects, explaining this difference by dose adjustment based on TBW (30).

Various studies have examined alternative weight descriptors for daptomycin dosing, challenging the use of TBW (34–38). Bhavnani et al. found that patients with TBW exceeding 110 kg are at a higher risk of elevated CPK levels when doses are based on TBW (5). In a study of 101 subjects, Fox et al. showed that clinical failure rates and safety were similar between patients receiving daptomycin based on AjBW or TBW (37). Finally, Olney et al. tested fixed daptomycin dosing and applied PK/PD methods to compare this approach to the current standard, concluding that fixed dosing, adjusted for sex and kidney function, is expected to improve the efficacy-to-toxicity ratio (38, 39). Thus, an alternative dosing strategy based on another body size metric, such as AjBW or IBW, may be safer for obese patients.

Known drug interactions with daptomycin are mainly related to an increased risk of muscular toxicity. Co-administration of statins is a well-established risk factor of such toxicity (40, 41). Other studies have suggested the role of other agents, such as antihistamines (17). Those interactions are thought to be pharmacodynamics only, without alteration of daptomycin PK.

In our study, the concomitant use of irbesartan was identified as a risk factor for daptomycin overexposure, which was not previously reported, to our knowledge. This did not appear to be a class effect since we observed no case in patients who were administered agents other than irbesartan (e.g., candesartan or losartan). Irbesartan was co-administered in approximately 20% of cases, compared with only 4% in controls. Univariate regression in the subgroup of subjects with normal renal function identified irbesartan as the most significant explanatory variable (*P* = 0.0018). Among the eight case subjects taking irbesartan, seven had CL_creat_ over 60 mL/min, suggesting that impaired renal function was not a confounder. Further analysis of PK parameters revealed a significant decrease in daptomycin clearance in patients receiving irbesartan, despite similar renal function. This suggests that irbesartan may reduce daptomycin clearance.

The mechanism of this potential interaction remains unclear. Up to 20% of an irbesartan dose is eliminated unchanged in the urine, and irbesartan is also a substrate of P-glycoprotein (P-gp), a renal efflux transporter (42, 43). Daptomycin is primarily eliminated by the kidneys, with 78% of the dose excreted unchanged. Some studies suggested that P-gp may influence daptomycin PK (10, 26, 44). One might hypothesize that an inhibition could occur, given that both drugs are P-gp substrates. Some studies have shown that irbesartan and telmisartan inhibit the transport of digoxin, a known P-gp substrate, but this inhibition was not observed for other angiotensin II receptor inhibitors (42, 43).

Another potential interaction pathway may involve OATP transporters, which are involved in the transport of angiotensin II receptor antagonists (45). Although daptomycin has not been identified as a substrate for these transporters so far, recent studies suggested that it may interact with other treatments through these same mechanisms (17). Nevertheless, these results should be interpreted with caution due to the limited number of patients taking irbesartan in our study. Further research is necessary to confirm this potential interaction and elucidate its mechanism.

This study has several limitations. Its retrospective, uncontrolled design limits the ability to establish causal relationships. The adherence to co-prescribing treatments could not be verified. Furthermore, as daptomycin concentrations were measured in routine TDM, potential inaccuracies in recording administration and sampling times, as well as assay errors, could have influenced the results. Overexposure definition was based on Cmin rather than AUC, because the latter is still not widely available in routine TDM. The threshold value of 60 mg/L defining overexposure was not based on safety events but rather corresponds to an unexpected, biological overexposure. The risk factors identified may have been different if another model or another threshold has been chosen. Nevertheless, a sensitivity analysis was performed for thresholds ranging from 50 to 70 mg/L and led to the same final model (data not shown). The trough concentrations, as well as the AUC in the case group (median = 2,460 mg·h/L, see Table 1), were much higher than values associated with increased risk of adverse events (Cmin > 24 mg/L or AUC > 939 mg·h/L). So, we think that the risk factors of overexposure identified are clinically relevant. However, further clinical studies should be performed to evaluate risk factors of toxicity.

The sample size was limited, with only 26 cases and 78 controls, which limits the statistical power and increases the risk of a type II error, or failure to detect a real effect. However, the use of multiple methods (descriptive analyses, regression analyses, and pharmacokinetic analyses) is a strength of the study, as it allows the results to be compared from three different perspectives and strengthens the conclusions.

### Conclusion

This case-control study confirms that renal impairment is the main risk factor of daptomycin overexposure. However, factors other than renal impairment may increase the risk of daptomycin overexposure, including high BMI and co-administration of irbesartan. In our study, the influence of BMI was attributed to the non-linear relationship between body weight and daptomycin PK parameters and the inappropriate use of weight-based dosing in patients with high BMI. The potential role of irbesartan needs to be confirmed in further studies. Our findings support the use of alternative body-size descriptors for daptomycin dosing in obese patients, such as AjBW or IBW. In addition, therapeutic drug monitoring may be of interest to control drug exposure and adjust dosage in individual patients with risk factors of overexposure.

## SUPPLEMENTAL MATERIAL

Supplemental material aac.01139-25-s0001.docx Tables S1 and S2; Fig. S1 to S4.

ASM does not own the copyrights to Supplemental Material that may be linked to, or accessed through, an article. The authors have granted ASM a non-exclusive, world-wide license to publish the Supplemental Material files. Please contact the corresponding author directly for reuse.
