# Supplementary material for: Risk factors of daptomycin overexposure: a case-control study
Source: Antimicrob Agents Chemother. 2025 Nov 7;69(12):e01139-25. doi: 10.1128/aac.01139-25 (PMC12691659; doi:10.1128/aac.01139-25)

**Risk factors of daptomycin overexposure: a case-control study**

**Supplementary material**

Clotilde Vellat^1,2^, Romain Garreau^1,2,3^, Aurélien Millet^4^, Catherine Piron^2^, Laurent Bourguignon^1,2,3^, Sandrine Roux^5^, Tristan Ferry^1,3,5^ and Sylvain Goutelle * ^1,2,3^ on behalf of the Lyon Bone and Joint Infection Study Group#

^1^ Laboratoire de Biométrie et Biologie Evolutive, UMR 5558, CNRS &Université Lyon 1, Villeurbanne, France.

^2^ Hospices Civils de Lyon, Groupement Hospitalier Nord, Service de Pharmacie, Lyon, France.

^3^ Univ Lyon, Université Claude Bernard Lyon 1, Facultés de Médecine et de Pharmacie de Lyon, Lyon, France.

^4^ Hospices Civils de Lyon, Groupement Hospitalier Sud, Service de Biochimie et Biologie Moléculaire, UM Pharmacologie -Toxicologie, Lyon, France.

^5^ Hospices Civils de Lyon, Groupement Hospitalier Nord, Hôpital de la Croix-Rousse, Service des Maladies Infectieuses et Tropicales, Centre de Référence pour la prise en charge des Infections Ostéo-Articulaires complexes (CRIOAc Lyon), Lyon, France.

**Table S1.** **Co-administered drugs in the case and control groups.**

| **ATC - Treatment classes** | **Case,**  **n = 26** | **Control,**  **n = 78** | **p-value²** | **q-value^3^** |
| --- | --- | --- | --- | --- |
| A02BA - H2-receptor antagonists | 0 | 1.3 | > 0.9 | > 0.9 |
| A02BC - PPI | 58 | 37 | **0.067** | 0.3 |
| A03A – Antispasmodics | 3.8 | 3.8 | > 0.9 | > 0.9 |
| A03F - Propulsives | 3.8 | 2.6 | > 0.9 | > 0.9 |
| A04A – Antiemetics | 7.7 | 3.8 | 0.6 | > 0.9 |
| A06A - Laxatives | 42 | 21 | **0.028** | 0.15 |
| A07 – Antidiarrheals agents | 3.8 | 3.8 | > 0.9 | > 0.9 |
| A09A – Digestives enzymes | 0 | 1.3 | > 0.9 | > 0.9 |
| A10A – Insulins | 12 | 10 | > 0.9 | > 0.9 |
| A10B – Blood glucose lowering drugs | 19 | 13 | 0.5 | > 0.9 |
| A11 – Vitamins | 38 | 33 | 0.6 | > 0.9 |
| B01AA – Vitamin K antagonists | 7.7 | 2.6 | 0.3 | 0.7 |
| B01AB – Heparin | 58 | 65.1 | > 0.9 | > 0.9 |
| B01AC – Platelet aggregation inhibitors | 35 | 19 | **0.11** | 0.4 |
| B01AF – Direct factor Xa inhibitors | 3.8 | 9 | 0.7 | > 0.9 |
| C01B – Antiarrhythmics | 7.7 | 2.6 | 0.3 | 0.7 |
| C01C – Cardiac stimulants | 0 | 1.3 | > 0.9 | > 0.9 |
| C02A – Other antihypertensives | 12 | 0 | **0.014** | 0.087 |
| C03A/C – Low/ Hight-ceiling diuretics | 42 | 21 | **0.028** | 0.15 |
| C03D – Potassium-sparing agents | 7.7 | 7.7 | > 0.9 | > 0.9 |
| C07 – Betablockers | 62 | 24 | **< 0.001** | 0.009 |
| C08A/D – Calcium channel blockers | 38 | 22 | **0.093** | 0.4 |
| C09A – ACE inhibitors | 27 | 15 | **0.2** | 0.7 |
| C09C – Angiotensin II receptor antagonists | 5 | 10 | **0.053** | **0.2** |
| C10AA – Statins | 12 | 12 | > 0.9 | > 0.9 |
| C10AB – Fibrates | 0 | 2.6 | > 0.9 | > 0.9 |
| C10AX – Other lipid-lowering drugs | 7.7 | 1.3 | **0.2** | 0.5 |
| D01A – Antifungals | 27 | 6.4 | **0.009** | 0.069 |
| D11A – Monoclonal antibodies | 0 | 1.3 | > 0.9 | > 0.9 |
| G04C / C02C – Antiadrenergic agents | 19 | 9 | **0.2** | 0.6 |
| H02A – Corticosteroids | 12 | 6.4 | 0.4 | > 0.9 |
| H03A – Thyroids | 23 | 2.6 | **0.003** | 0.029 |
| J01A – Tetracyclines | 0 | 9 | **0.2** | 0.6 |
| J01C – Beta-lactams antibacterials | 42 | 41 | > 0.9 | > 0.9 |
| J01E – Sulfonamides and trimethoprim | 0 | 6.4 | 0.3 | 0.8 |
| J01F – Macrolides | 0 | 6.4 | 0.3 | 0.8 |
| J01M – Quinolone antibacterial | 15 | 18 | > 0.9 | > 0.9 |
| J01X – Glycopeptide antibacterials | 0 | 3.8 | 0.6 | > 0.9 |
| J01X – Other antibacterials | 15 | 17 | > 0.9 | > 0.9 |
| J05A – Antivirals | 0 | 2.6 | > 0.9 | > 0.9 |
| L04A – Immunosuppressants | 3.8 | 1.3 | 0.4 | > 0.9 |
| M01A – NSAID | 0 | 5.1 | 0.6 | > 0.9 |
| M03B – Muscle relaxants | 3.8 | 2.6 | > 0.9 | > 0.9 |
| M04A – Antigout | 12 | 7.7 | 0.7 | > 0.9 |
| N02A – Opioids analgesics | 50 | 55 | 0.6 | > 0.9 |
| N02B – Non-opioids analgesics | 81 | 71 | 0.3 | 0.8 |
| N03A – Antiepileptics | 23 | 26 | 0.8 | > 0.9 |
| N04B – Anti-Parkinson drugs | 3.8 | 0 | 0.3 | 0.8 |
| N05A – Antipsychotics | 12 | 7.7 | 0.7 | > 0.9 |
| N05B – Other anxiolytics | 0 | 2.6 | > 0.9 | > 0.9 |
| N05B – Benzodiazepine derivatives | 19 | 22 | 0.8 | > 0.9 |
| N05C – Benzodiazepines related drugs | 19 | 14 | 0.5 | > 0.9 |
| N06AA – NSMRI | 3.8 | 6.4 | > 0.9 | > 0.9 |
| N06AB – SSRI | 7.7 | 7.7 | > 0.9 | > 0.9 |
| N06AX – Other antidepressants | 7.7 | 6.4 | > 0.9 | > 0.9 |
| R06A – Antihistamines | 19 | 14 | 0.5 | > 0.9 |
| V03A – Hyperkalemia treatments | 12 | 0 | **0.014** | 0.087 |
| 1- All values are given in %.  2 – Pearson’s Chi-squared test or Fisher’s exact test.  *PPI*, Proton pump inhibitors; *ACE*, angiotensin-converting enzyme; *NSAID*, non-steroid anti-inflammatory; *SSRI*, Selective serotonin reuptake inhibitors; *NSMRI*, Non-selective monoamine reuptake inhibitors. | | | | |

**Table S2.** **Parameters of the population pharmacokinetic model used in data analysis** (model previously published by Garreau et al. <https://doi.org/10.1093/jac/dkab006>)

| Parameters | Final estimate | Relative standard error (%) |
| --- | --- | --- |
| Fixed effects |  |  |
| CL_DAP_ (L/h) | 0.365 | 4.1 |
| θ1 _(CL, CLCR)_ | 0.43 | 8.4 |
| θ2 _(CL, Sex)_ | 0.232 | 16.5 |
| V1 (L) | 3.59 | 9.7 |
| θ3 _(V1, Age)_ | 0.263 | 25.3 |
| θ4 _(V1, WT)_ | 0.603 | 12.2 |
| θ5 _(V1, Rif)_ | -0.121 | 42 |
| θ6 _(V1, Sex)_ | 0.117 | 32.7 |
| Q (L/h) | 0.752 | 11.2 |
| V2 (L) | 4.71 | 9.8 |
|  |  |  |
| Random effects |  |  |
| ω_CL_ | 0.196 | 9 |
| ω_V1_ | 0.123 | 21 |
| ω_Q_ | 1.13 | 11.7 |
| ω_V2_ | 0.29 | 23.9 |
| γ_CL_ | 0.193 | 4.5 |
| γ_V1_ | 0.24 | 11.4 |
| Abbreviations: CL_DAP_, daptomycin clearance; CL_CR_, creatinine clearance; Q, intercompartment clearance; V1, central volume of distribution; V2, peripheral volume of distribution.  The relationships between individual parameters (CL_DAP(i)_ & V_1(i)_) and covariates were as follows: CL_DAP(i)_ = CL_DAP(_* exp[θ1 × (CL_CR_/109) + θ2 * (if male)] * exp(η_i_) * exp(κ_ij_),  where 109 is the median CL_CR_ in the population.  V1_(i)_ = V1 * exp[θ3 * (Age/60.4) + θ4*(WT/79.2) + θ5 (if rifampicin co-administration) + θ6 (if male)] * exp(ηi) * exp(κij), where 60.4 and 79.2 are the median values of age (in years) and weight (in kg) in the study population, respectively. | | |

**Figure S1.** **Cumulative probability of daptomycin steady state trough concentration at 24h**

Results were computed for a typical individual of 70 kg with normal renal function.

The black line represents the median value, dashed lines represent the 5th and 95th percentiles, and the red line represents the selected overexposure threshold.

*
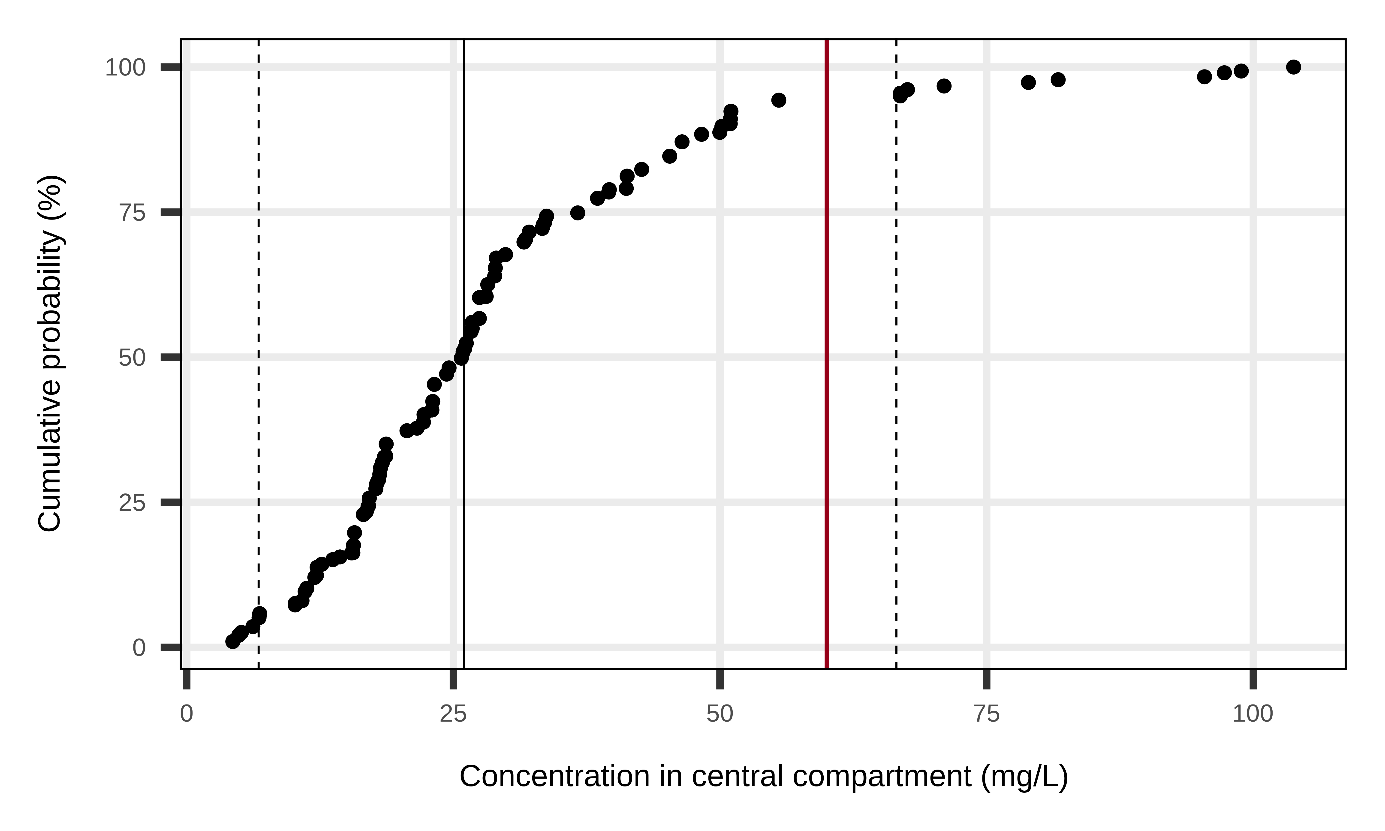
*

**Figure S2.** **Variable Importance Plot of the logistic regression model.**


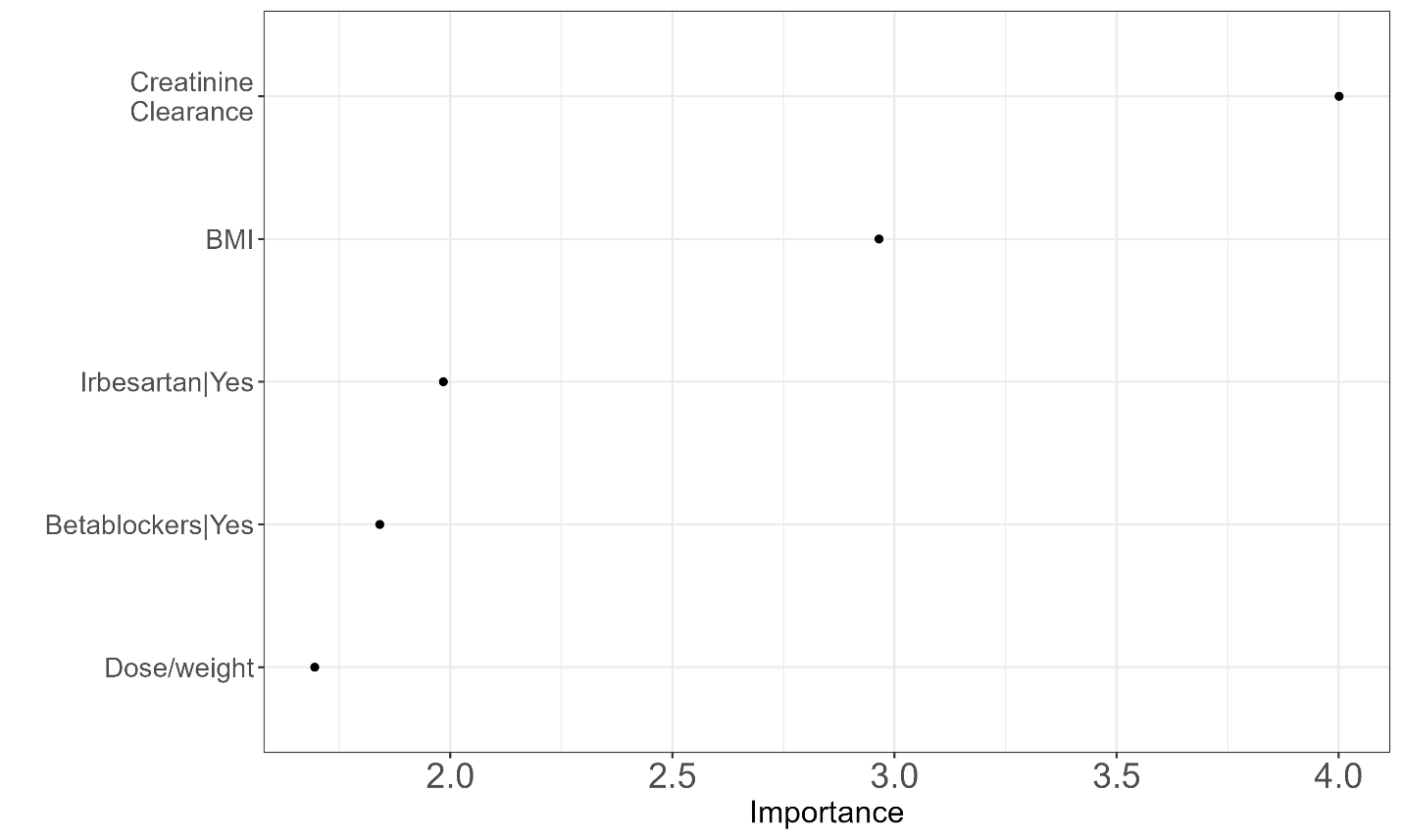


**Figure S3**. **Precision Recall Curve for classification by the logistic regression model.**


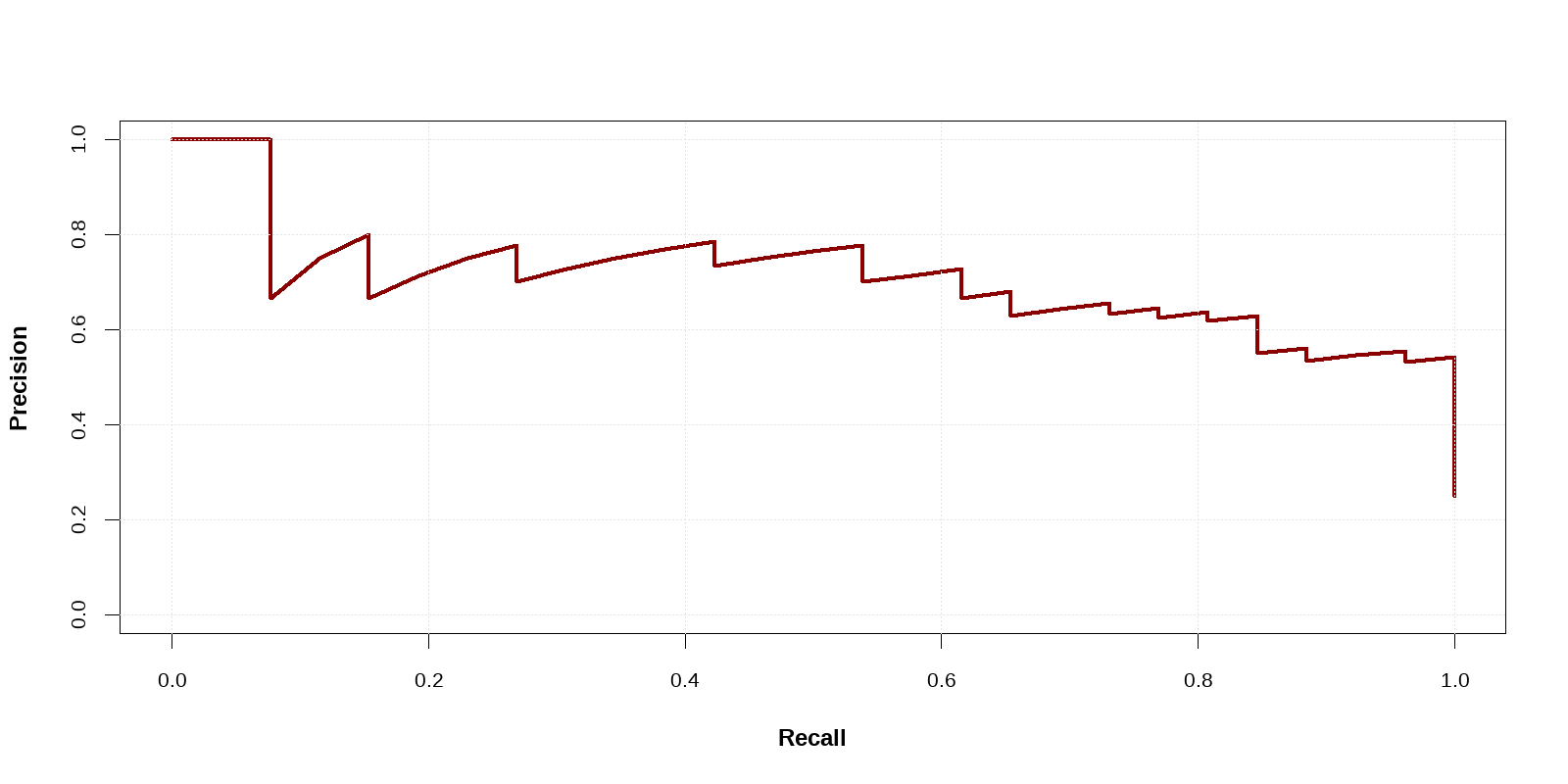


**Figure S4.** **Observed daptomycin trough concentrations according to angiotensin II receptor blockers co-administration.**

The dashed black line represents the overexposure threshold considered in this study.


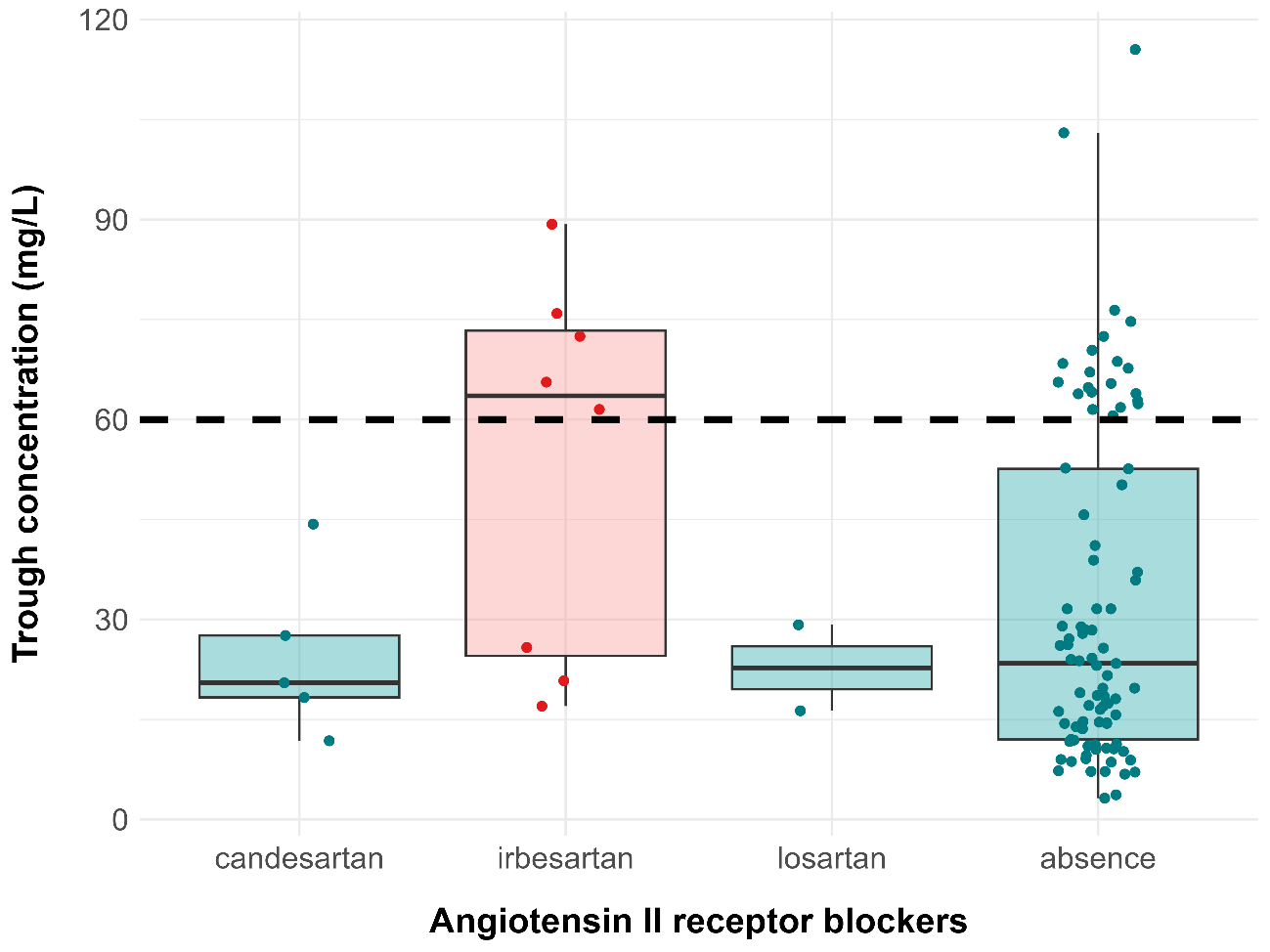

Supplement: Supplemental material — Tables S1 and S2; Fig. S1 to S4. [file aac.01139-25-s0001.docx]
